# Draft Genome Sequence of a Dictyoglomus sp. from an Enrichment Culture of a New Zealand Geothermal Spring

**DOI:** 10.1128/genomeA.00150-18

**Published:** 2018-03-15

**Authors:** Anna-Louise Reysenbach, John A. Donaho, John F. Kelley, Emily St. John, Christina Turner, Mircea Podar, Matthew B. Stott

**Affiliations:** a Biology Department, Portland State University, Portland, Oregon, USA; b Oak Ridge National Laboratory, Oak Ridge, Tennessee, USA; c School of Biological Sciences, University of Canterbury, Christchurch, New Zealand

## Abstract

A draft genome of a novel Dictyoglomus sp., NZ13-RE01, was obtained from a New Zealand hot spring enrichment culture. The 1,927,012-bp genome is similar in both size and G+C content to other Dictyoglomus spp. Like its relatives, Dictyoglomus sp. NZ13-RE01 encodes many genes involved in complex carbohydrate metabolism.

## GENOME ANNOUNCEMENT

Dictyoglomus spp. form a distinct bacterial phylum, with only two described species with genome sequences, D. turgidum (1) and D. thermophilum (2, 3). Dictyoglomus spp. have been detected in thermophilic environments, such as terrestrial hot springs (2, 4–8) and paper pulp factory effluent (9), and they have an unusual morphology consisting of large spherical bodies assembled from bundles of filamentous cells (2, 5, 9). Dictyoglomus spp. are of industrial interest due to their capability to ferment a wide range of complex carbohydrates, including starch, cellulose, xylan, and pectin (2, 8, 10, 11). Due to the low number of representative genomes, their phylogenetic placement within the domain Bacteria is not certain as *Dictyoglomus* spp. are, although they appear to be affiliated most closely with a cluster containing Coprothermobacter, Synergistes, Acetothermia, and Thermotogales (1, 12, 13).

We obtained a genome of a novel Dictyoglomus sp., NZ13-RE01, from a New Zealand hot spring enrichment culture (Hell’s Gate, Tikitere, New Zealand, 38°03′47″S, 176°21′39″E, pH 6.0, 74°C). Enrichments were incubated at 80°C for 4 days in a modified anaerobic DSMZ medium (no. 88) containing yeast extract (0.5 g/liter) and tryptone (0.5 g/liter). Spherical bodies were apparent in the enrichment cultures by phase-contrast microscopy. DNA was extracted using the Qiagen DNeasy blood and tissue kit. Metagenome Nextera DNA libraries were sequenced on the Illumina MiSeq platform. Adapters and low-quality reads were trimmed using Trimmomatic (14), reads were assembled with IDBA-UD version 1.1.0 (15, 16), and contigs ≥1 kb were binned using MaxBin version 1.4.5 (17). To optimize the assembly, the reads were mapped back to the Dictyoglomus sp. NZ13-RE01 draft genome using Bowtie2 version 2.2.5 (18) and SAMtools version 1.2 (19, 20) and reassembled with IDBA-UD. The genome was further curated using emergent self-organizing maps (21). Open reading frames were annotated using the Rapid Annotations using Subsystems Technology (RAST) server (22–24), the NCBI Prokaryotic Genome Annotation Pipeline (PGAP) (25), the Clusters of Orthologous Groups of proteins (COG) database (26), and the dbCAN database (27). tRNAs were predicted with tRNAscan-SE version 2.0 (28).

Based on CheckM (29) analysis, the Dictyoglomus sp. NZ13-RE01 genome is about 100% complete with no contamination. The 1,927,012-bp genome consists of 34 contigs, with a 33% G+C content, 1,870 predicted protein-coding genes, and 48 tRNAs. The Dictyoglomus sp. NZ13-RE01 genome has an average nucleotide identity (ANI) score of 74% and an average amino acid identity (AAI) score of 65% compared to the genomes of both D. turgidum and D. thermophilum (30, 31). Further, Circos synteny plots (32) show that the Dictyoglomus sp. NZ13-RE01 genome does not share the highly syntenic genome arrangement found between D. turgidum and D. thermophilum (1). Considering the dissimilarity in genome nucleotide and amino acid sequences, and the low synteny in genome arrangement between NZ13-RE01 and either of the described Dictyoglomus spp., it is likely that NZ13-RE01 represents a new species within the genus Dictyoglomus.

The Dictyoglomus sp. NZ13-RE01 genome encodes an extensive suite of carbohydrate metabolism genes (63 glycosyl hydrolases, 10 carbohydrate esterases, and 28 glycosyltransferases), including α-amylases, α-xylosidases, a chitinase, endo-1,4-β xylanases, and a β-mannanase. Like other Dictyoglomus spp., the genome has a reverse gyrase. The sporulation gene, *spoVS* , was also present and has been proposed to play a role in morphological changes in Dictyoglomus spp. (1).

### Accession number(s).

The nucleotide genome sequence reported here has been deposited in DDBJ/ENA/GenBank under the accession no. NIRF00000000. The version described in this paper is the first version, NIRF01000000.
